# Colonic Intussusception Due to a Cecal Tumor: A Representative Case

**DOI:** 10.7759/cureus.36338

**Published:** 2023-03-18

**Authors:** Grant Hubbard, Keaton Wood, Lahari Vudayagiri, Hannah Chong, Rick Gemma

**Affiliations:** 1 General Surgery, Western Reserve Hospital, Cuyahoga Falls, USA; 2 General Surgery, Rocky Vista University College of Osteopathic Medicine, Greenwood Village, USA

**Keywords:** mechanical large bowel obstruction, gi oncology, colonic intussusception, emergent general surgery, invasive colon cancer

## Abstract

We present a case of ileo-colic intussusception in a 58-year-old female, with representative clinical features and useful intraoperative images. These cases are relatively rare in adults and should always be concerning for underlying malignancy, as was seen in our patient's case. In recent years, there has been a slight shift in the management of this pathology, and we present our arguments in agreement with these changes.

## Introduction

The phenomenon of bowel intussusception was first described by Barbette in 1789 as a proximal portion of the intestine (intussusceptum) telescoping into a distal portion of the intestine (intussuscipiens) [1]. However, it was not until Hutchinson in 1871 that the first reduction would be described [2]. By far, intussusception is more common in children, with likely etiologies being Meckel's diverticulum and hyperplastic lymphoid tissue after gastrointestinal virus exposure [3]. Intestinal intussusceptions are rarer in adults, representing only 1%-5% of intestinal obstructions, often leading to the discovery of an organic cause, which may be neoplastic [4]. Segments of the enteric tract that is theoretically more susceptible to intussusception are junctions between freely moving segments and fixed segments, such as the ileum or cecum into the ascending colon. Intussusceptions are classified according to etiology (idiopathic, primary, or secondary) and by their locations (entero-enteric, ileo-colic, colo-colic, etc.) [3,4].

In adults, approximately one-third of intussusceptions are due to an idiopathic mechanism [5]. Adult intussusceptions more often occur in the small intestine. Of the remaining 80% of cases, there is a secondary cause from a benign or malignant tumor of the bowel wall or an irritant within the lumen that triggers inflammation, which alters peristalsis [5,6]. In some reported series, malignancy accounts for up to 30% of small intestine intussusceptions and 66% of large bowel intussusceptions [3]. Given the rarity in adults and the multitude of clinical presentations, the exact diagnosis and identification of the intussusceptum/intussuscipiens can be difficult to determine, even with high-quality cross-sectional imaging [6]. Other studies report attempting endoscopy to locate the lesions and the corresponding lead points, which may be able to identify ileo-colic, colo-colic, and sigmoid-rectal intussusceptions [5]. Colonoscopy has the added benefit of providing the opportunity for therapeutic reduction [3], but this is not always possible. In the majority of cases in adults, surgery remains the optimal management strategy for the reduction and resection of any underlying neoplastic process [4-6].

## Case presentation

**Figure 1:**
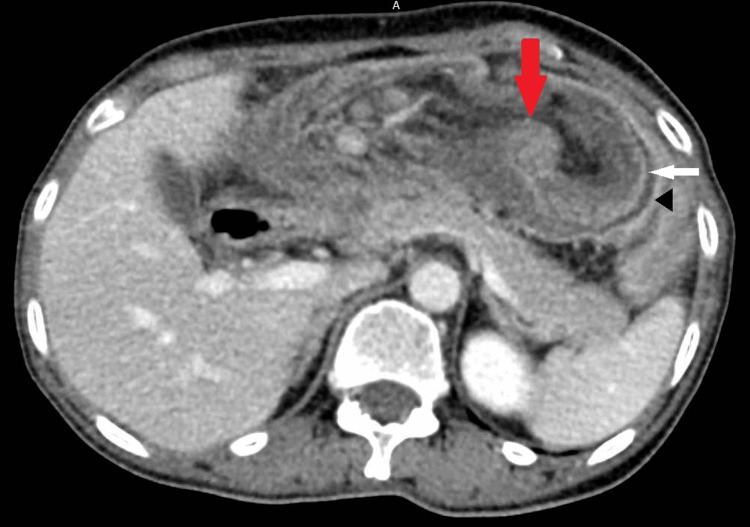
Representative image from preoperative CT scan showing the cecum intussuscepted into the colon. The red arrow points to the cecal tumor. The white arrow points to the cecal bowel wall, intussuscepted up to the splenic flexure (noted by the black arrowhead). CT: computed tomography

The patient is a 58-year-old female who presented to the emergency department with a five-day history of cramping and sharp right-sided abdominal pain. Medical history was notable only for thyroidectomy due to nontoxic multinodular goiter and hepatitis B in the remote past. She described intermittent episodes of cramping abdominal pain and nausea throughout the preceding year, as well as a weight loss of 10 lbs. These episodes of pain would typically last for 2-3 days and then spontaneously resolve. She denied changes in her stool appearance or caliber. She was a Vietnamese immigrant to the United States approximately five years prior to presentation. She denied any significant family history of abdominal cancers. On initial examination, she was tachycardic to 114 beats per minute (bpm), with the rest of her vital signs being stable. Physical examination was only notable for generalized abdominal tenderness to deep palpation and the presence of moderate distension, but no rebound tenderness or guarding was noted. Laboratory evaluation on presentation was unremarkable; results showed no prominent leukocytosis or lactic acidosis. A computed tomography (CT) scan of the abdomen and pelvis with intravenous contrast demonstrated a long segment of proximal and transverse colo-colic intussusception, with significant resultant small bowel dilation (Figure 1). Some nonspecific edema was noted in the small bowel mesentery due to this process. Consequently, she had a nasogastric tube placed for enteric decompression, and plans were made for operative intervention.

Differential diagnosis at this point included large bowel intussusception, malignant obstruction of the large bowel, mesenteric ischemia or ischemic colitis, and gastroenteritis. Intussusception was felt to be the most likely diagnosis due to the appearance of the bowel on the CT scan, in combination with the patient's description of intermittent cramping abdominal pain. Because an intussusception in this patient population can be due to a benign (i.e., lipoma and polyp) or malignant (i.e., adenocarcinoma) process, we opted to approach the surgery with the assumption that the intussusception was due to a malignant process. A large bowel obstruction in this patient would most likely be due to a malignant process; however, the management pathway for this would still lead to surgery, for either resection or diverting ostomy. In light of that, the decision was maintained to proceed with surgery. An ischemic process such as mesenteric ischemia or ischemic colitis was felt to be possible but unlikely and did not warrant further dedicated workup with CT angiography or ultrasonography.

**Figure 2:**
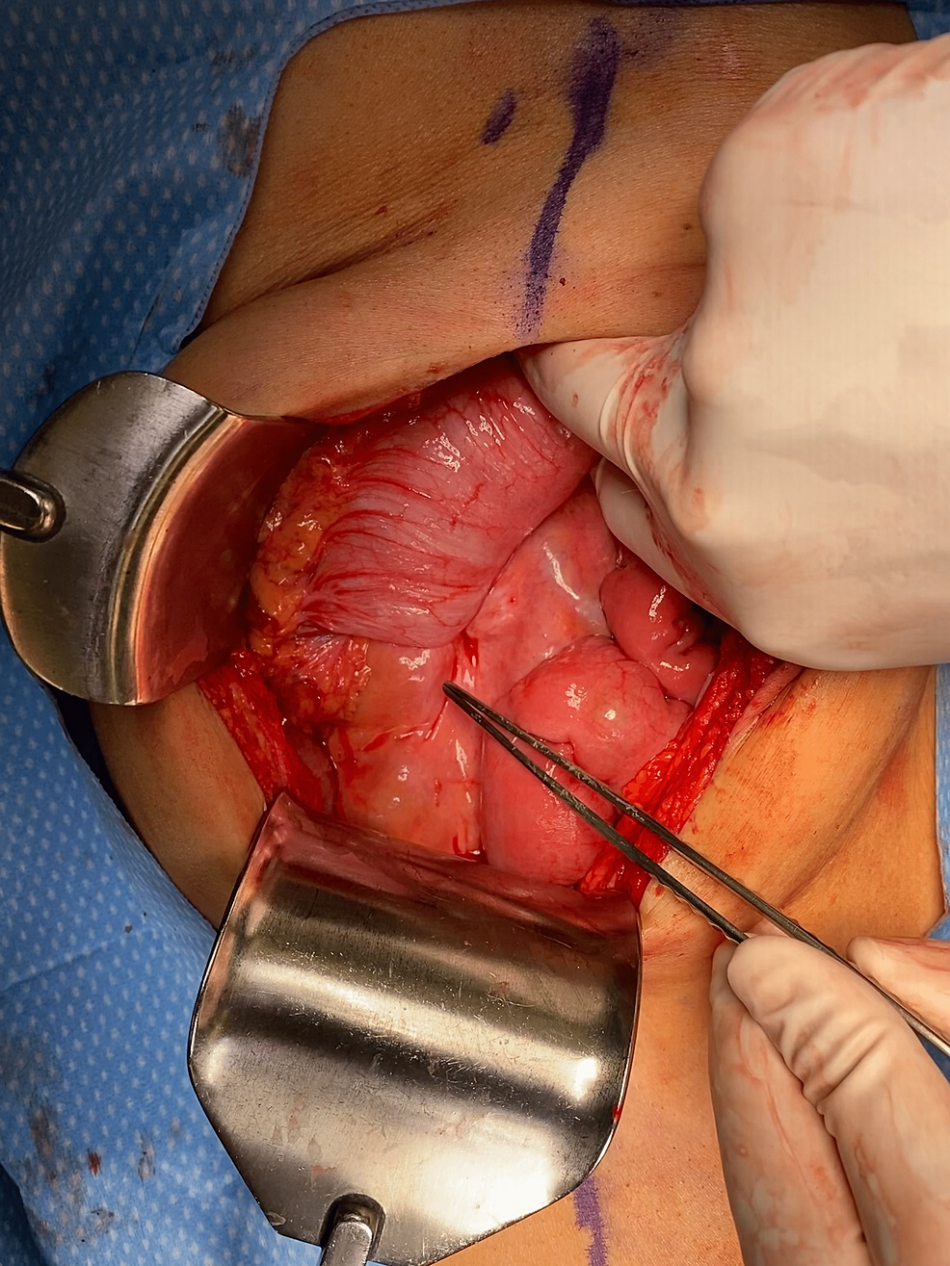
The cecum and terminal ileum intussuscepted into the ascending and transverse colon. The instrument points to the small bowel extruding from the right colon (intussusceptum). The surgeon's right hand is on the right colon (intussuscipiens).

**Figure 3:**
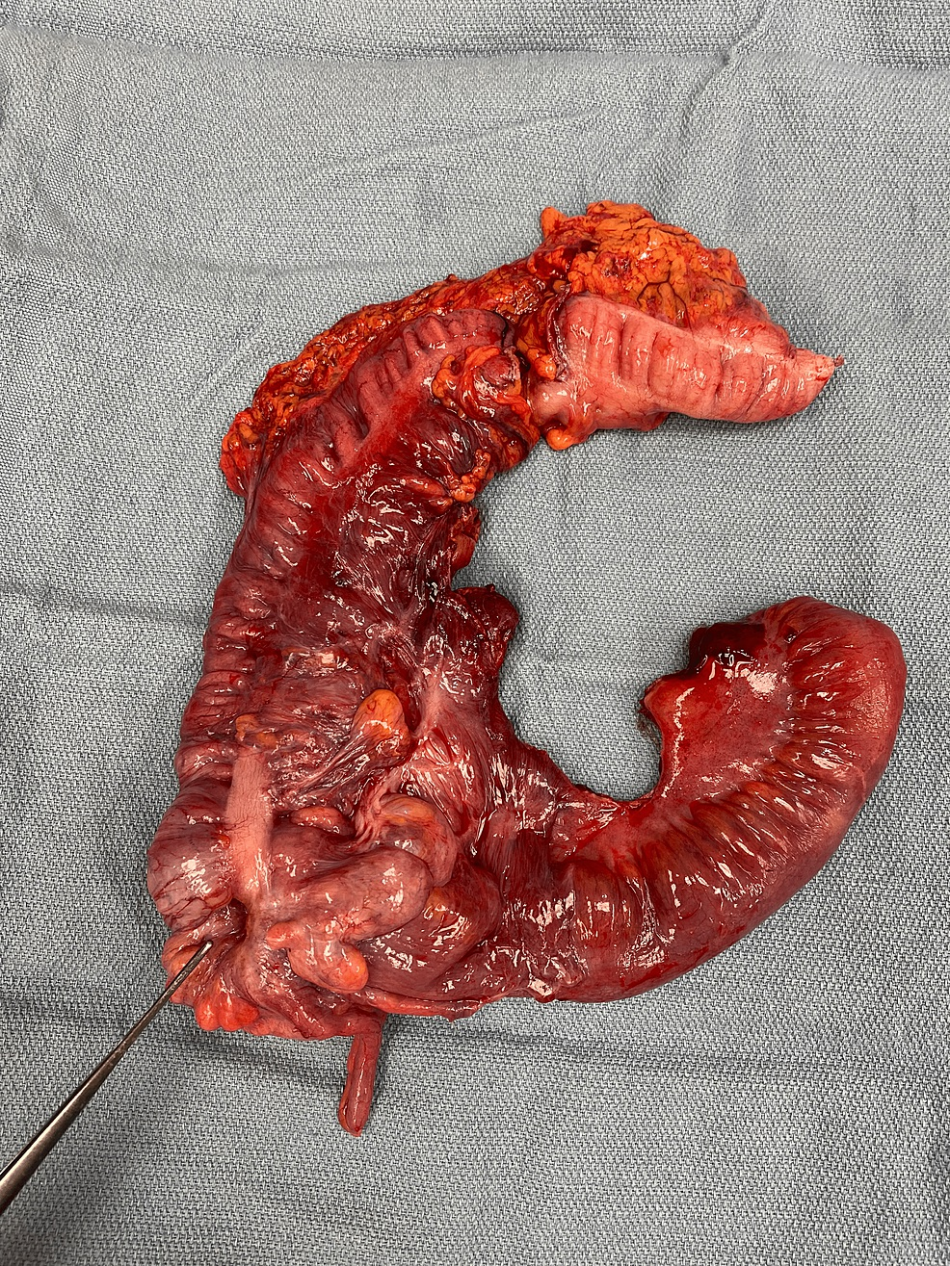
Resected specimen of the terminal ileum through the proximal transverse colon. The instrument points to the cecal adenocarcinoma, which served as the lead point for the intussusception.

The patient was taken to the operating room for an exploratory laparotomy. Intraoperatively, the intussusception was identified involving the terminal ileum up to the transverse colon (Figure 2); the lead point migrated to the mid-transverse colon. The intussusception was reduced, after which an obvious 3 cm fungating mass (Figure 3) was identified at the cecum, which had served as the lead point for the intussusception. A right hemicolectomy was performed with stapled ileo-colic anastomosis. Attention was paid to performing a high ligation of the mesenteric pedicle, to maximize lymph node yield for histologic evaluation.

The patient recovered well after surgery, tolerated an oral diet, and was ultimately discharged home on postoperative day 7. Pathology showed pT2, N0, M0, invasive moderately differentiated adenocarcinoma with mucinous features involving the muscularis propria, with 16 evaluated nodes negative for carcinoma. Additional CT of the chest confirmed no distantly metastatic disease, diagnosing her with a stage 1 colon cancer.

The tumor was *MLH-1 *positive, *MSH-2 *positive, *MSH-6 *negative, and *PMS-2 *negative, a sequence suggestive for the inheritance of a loss of DNA mismatch repair proteins, commonly seen in inherited syndromes such as Lynch syndrome, also known as hereditary nonpolyposis colorectal cancer (HNPCC) syndrome. Further genetic testing demonstrated a *PMS-2*-related Lynch syndrome. After additional counseling, the patient opted for aggressive routine screening with endoscopy and frequent physical examinations, rather than prophylactic subtotal colectomy. She recovered well and returned to her everyday routines without any symptomatic complaints.

## Discussion

Our report demonstrates a representative case of ileocecal intussusception. An intussusception as the initial identification of cecal cancer is uncommon but has been reported. In a series of adult patients from a single center in Japan by Honjo et al. [2], 44 patients were identified over 16 years, which correlated to a prevalence of 0.2% among all of their patients. They reported that six of their adult intussusceptions (13%) involved a cecal tumor intussuscepting into the ascending colon, as was seen in our case. Similar results were seen in an older retrospective review out of China by Wang et al. [5].

The presentation of intussusception with benign or malignant lead points is similar, in both symptomatology and imaging characteristics [6]. The primary presenting symptom is abdominal pain (in 80%-90% of patients), which is characterized as intermittent and crampy in 20%-30% of patients [6]. It is unclear whether this pain is due to the actual intussuscepted tissue or the resulting bowel obstruction. Our patient likely had been intermittently intussuscepting over the year prior to presentation, with resultant obstructions from this that would resolve as soon as the intussusception had reduced. Her ultimate obstruction that brought her into our emergency room was the most exaggerated of these episodes.

It is our opinion that abdominopelvic CT scans provide a reliable way of preoperatively diagnosing an intussusception and identifying the involved segments. Elsewhere in the literature, this opinion has been corroborated, with up to 100% of cases in some series accurately diagnosed on CT scan [2,3]. Ultrasound can be useful but generally has inferior sensitivity and specificity. Plain film radiographs have limited to no utility. On abdominal examination, the intussusceptum may be felt as a palpable mass, but the absence of a mass on examination should not be used to rule out the diagnosis of intussusception.

Authors since the mid-1950s have argued in favor of an en bloc resection without reduction, especially when malignancy is suspected as the lead point for the cancer [1,5,7]. The primary concern that necessitated this approach was the fear that the handling of the bowel malignancy would cause venous translocation of tumor cells or seeding into the abdomen. However, more recently, the approach of the reduction of the intussusception prior to resection, either endoscopically or surgically, has been advocated [2,3,6]. This is particularly useful when there is a long segment of intussusception so that resection can be more limited than a complete en bloc resection would require [6]. In our experience with this patient and others, we have found success with operative reduction prior to resection. We have not seen cases result in distant metastasis or intra-abdominal seeding from handling the tumor.

This patient's eventual diagnosis of Lynch syndrome is certainly a unique pathologic finding, but this ultimately did not play into her development of intussusception.

## Conclusions

Adult intussusception is an uncommon diagnosis but an important one to identify due to its association with malignancy. In this report, we have presented a representative case that serves as a useful learning tool to identify common findings for these patients and how to manage them. We have also provided figures that provide a solid identification of the anatomy relevant to an intussusception.
